# The Effects of Galantamine Hydrobromide Treatment on Dehydroepiandrosterone Sulfate and Cortisol Levels in Patients with Chronic Fatigue Syndrome

**DOI:** 10.4306/pi.2009.6.3.204

**Published:** 2009-06-23

**Authors:** Tayfun Turan, Hasan Basri Izgi, Saliha Ozsoy, Fatih Tanrıverdi, Mustafa Basturk, Akif Asdemir, Aslı Beşirli, Ertugrul Esel, Seher Sofuoglu

**Affiliations:** 1 Department of Psychiatry, Erciyes University Medical School, Kayseri, Turkey.; 2 Department of Endocrinology, Erciyes University Medical School, Kayseri, Turkey.

**Keywords:** Chronic fatigue syndrome, Cortisol, Dehydroepiandrosterone sulfate, Galantamine hydrobromide

## Abstract

**Objective:**

Mental fatigue, cognitive disorders, and sleep disturbances seen in chronic fatigue syndrome (CFS) may be attributed to cholinergic deficit. A functional deficiency of cholinergic neurotransmission may cause the hypothalamic-pituitary-adrenal axis hypoactivity seen in CFS. Therefore, we investigated the alterations in stress hormones such as cortisol and dehydroepiandrosterone sulfate (DHEAS) in CFS patients before and after 4-week administration of galantamine hydrobromide, a selective acetylcholinesterase inhibitor, and aimed to investigate whether there are any relationships between the probable hormonal changes and cholinergic treatment.

**Methods:**

Basal levels of cortisol and DHEAS were measured in 29 untreated CFS patients who were diagnosed according to Centers for Disease Control (CDC) criteria and in 20 healthy controls. In the patient group, four weeks after 8 mg/d galantamine hydrobromide treatment, cortisol and DHEAS levels were measured again. After the treatment 22 patients who stayed in study were divided into two subgroups as responders and nonresponders according to the reduction in their Newcastle Research Group ME/CFS Score Card (NRG) scores.

**Results:**

Important findings of this study are lower pre-and post-treatment cortisol levels and in all CFS patients compared to controls (F=4.129, p=0.049; F=4.803, p=0.035, respectively); higher basal DHEAS values and higher DHEAS/cortisol molar ratios which were normalized following four weeks' treatment with 8 mg/d galantamine hydrobromide in the treatment-respondent group (F=5.382, p=0.029; F=5.722, p=0.025, respectively).

**Conclusion:**

The findings of the decrease in basal DHEAS levels and DHEAS/cortisol molar ratios normalizing with galantamine treatment may give some support to the cholinergic deficit hypothesis in CFS.

## Introduction

Diagnostic criteria for chronic fatigue syndrome (CFS) include lymphadenopathy, sore throat, cognitive impairment, headache, muscle and joint pains and tiredness following activity in addition to fatigue that lasts for more than six months, leading to disability and that does not improve with rest.^1^ Psychiatric co-morbid conditions like depression and anxiety are present in almost 50% of patients.^2^ These co-morbid conditions complicate the evaluation of endocrine disorders like dysfunction in the hypothalamic-pituitary-adrenal (HPA) axis.

The etiology of CFS is not clear. However, accumulating data suggest that underlying pathophysiology of stress related disorders such as CFS involves alterations in the reactivity of HPA axis response to physical and psychosocial stress and as a consequence it might cause a HPA axis switch from hyper-to hypofunction position.^3^ Hypocortisolism, which appears as a cardinal feature of CFS without psychiatric comorbidity, does not seem to be explained by only inactivity.^4^,^5^ The presence of hypocortisolism in CFS has been shown by low cortisol levels in 24-hour urine,^6^-^8^ by low serum cortisol levels early in the morning,^9^,^10^ and by low saliva cortisol levels.^11^

Recently, dehydroepiandrosterone (DHEA) produced in the zona reticularis of the adrenal cortex and its more stable sulfate derivative (dehydroepiandrosterone sulfate, DHEAS) became the focus of attention in addition to glucocorticoids produced in the zona fasciculate in CFS. DHEA and DHEAS are neurosteroids that are also produced in the brain and their release from adrenal cortex is under the control of adrenocorticotrophic hormone (ACTH).^12^ They have a diurnal rhythm similar to cortisol, that is to say their maximum release is early in the morning just before awakening.^13^,^14^ Plasma levels of DHEAS are more stable than DHEA and may be a better measure of adrenal androgen turnover.^15^

Parker et al.^16^ proposed that there was a symbiosis between DHEA and cortisol production and that this production shifted from androgens to glucocorticoids for homeostasis during psychological or physical stresses. However, in CFS, which is a stress-related disorder, the metabolic shift expected from DHEA to cortisol was not observed during physiological stress.^17^ Another study showed impaired DHEA responsiveness to stimulation with synthetic ACTH in CFS patients.^18^

Studies conducted in both animals and humans demonstrate positive effects of DHEA and DHEAS on memory (promnestic effects) by increasing acetylcholine release from the hippocampus.^19^,^20^ Its levels are found to be low in conditions associated with memory impairment.^21^ Mental fatigue and cognitive disorders in CFS may be attributed to cholinergic deficit. Acetylcholine has a general steroidogenic effect and this effect is mediated by ACTH via muscarinic receptors.^22^-^24^ Under conditions of stress, acetylcholine facilitates the release of corticotropin-releasing hormone (CRH). A functional deficiency of cholinergic neurotransmission may cause the HPA axis hypoactivity seen in CFS since cholinergic activity potentially stimulates the HPA axis.^25^ Sleep disturbances are common in patients with CFS and may be due to a central cholinergic deficiency.^26^ Galantamine hydrobromide is a selective acetylcholinesterase inhibitor. In a randomized controlled study it was found that galantamine did not have any therapeutic effect in CFS patients.^26^ In another uncontrolled study, it was administered to CFS patients with slight psychiatric features for two weeks at a dose of 10 mg/d, and it was reported that fatigue, sleep disorder, and myalgia symptoms improved significantly.^27^ There have been few studies evaluating the effect of galantamine on cortisol levels. The findings of these studies are inconsistent. Although a study showed increased cortisol levels by galantamine,^28^ in a recent study no galantamine treatment effect on cortisol levels was demonstrated.^26^

In this study, we investigated probable alterations in stress hormones such as cortisol and DHEAS in CFS patients before and after the treatment with galantamine hydrobromide.

## Methods

### Subjects

Twenty-nine CFS patients who were diagnosed according to Centers for Disease Control (CDC)^1^ criteria (20 females and 9 males; mean age: 41.89±8.53 years; mean disease duration: 80.13±57.16 months) and 20 healthy controls (16 females and 4 males; mean age: 42.50±9.42 years) were included in the study. The patients were selected consecutively from the outpatient population of the Psychiatry Clinic of Erciyes University Medical School. None of the patients was or had ever been diagnosed with any psychiatric disease according to Diagnostic and Statistical Manual of Mental Disorders, 4th Edition, Text Revision (DSM-IV TR) criteria.^29^ The control group included healthy hospital personnel who did not have any psychiatric disease history or diagnosis. Medical disorders (e.g. neurological, endocrinological, or metabolic) were excluded through the history, clinical examinations, and the evaluation of the results of the laboratory tests in all subjects. None of the subjects was using any medication or oral contraceptive drug that could affect the functions of the HPA axis. The female subjects were all pre-menopausal.

This study was approved by the local Ethics Committee. Written informed consent was obtained from each subject after the study was explained to them.

### Procedure

In the patients the first stage involved a standard clinical evaluation and the administration of the following clinical scales: the Hamilton Depression Rating Scale (HAM-D);^30^ Hamilton Anxiety Rating Scale (HAM-A);^31^ and Newcastle Research Group ME/CFS Score Card (NRG),^32^ which consists of 10 items and measures the physical and cognitive functions, and on which a score of ≥15 is highly suggestive of CFS. After this, catheters were placed in the forearm veins of the subjects at 8.00 am after a starvation period of 12 hours and, following a resting period of 30 minutes, blood samples were collected into tubes. The samples were centrifuged directly Separated serum samples were stored at -70℃. Basal blood samples of the female patients were obtained between the 4th and 7th days of their menstrual cycles. Then, all patients were started on fixed dose 8 mg/d oral galantamine hydrobromide. After four weeks of treatment, the same psychiatric measures and blood sampling procedure were repeated in the patients. However, seven patients were later excluded as they were unable to tolerate the treatment because of nausea or would not agree to cooperate, and the study continued with 22 patients. The same psychiatric measures and blood taking procedure for hormonal values were carried out only once in healthy controls.

At the end of the treatment period we separated our patients into two subgroups: responders (n=10) and non-responders (n=12). Twenty percent and more reductions in NRG scores were accepted as a response to treatment arbitrarily.^32^

### Hormone analyses

Serum cortisol levels [{Diagnostic System Laboratories (DSL, Webster, TX, USA) DSL-2100 reagents}, (sensitivity: 0.3 µg/dL, coefficient of variation: 8.4)] and DHEAS levels [{DSL (Webster, TX, USA) DSL-3500 reagents}, (sensitivity: 17.0 ng/mL, coefficient of variation: 6.3)] were measured by the radioimmunoassay (RIA) method.

### Statistical analyses

Data distributions for each hormonal evaluation were normal according to the Kolmogorov-Smirnov test. The patients and controls were compared with respect to sex using the χ^2^ test. The significance of differences between age, body mass index (BMI), and clinical scale scores of the patients and controls was assessed with a t-test for independent groups. To compare hormone levels of the patients and controls a two-way analysis of variance (ANOVA) test was performed by taking the presence of disorder and gender as fixed factors, and age and BMI as covariates. A repeated measure ANOVA test was carried out for the comparison of the hormone levels of the patients before and after the treatment. The same tests were used for analyses of subgroups according to the response to the treatment (responders and non-responders). Pearson's correlation test was performed to investigate the relationships between hormone levels and clinical and demographical variables in the patients and controls. The changes in hormonal values and those in clinical scales were calculated as delta values by subtracting the post-treatment values from the pre-treatment values.

## Results

No statistically significant differences were observed between the two groups according to age, gender, or BMI variants. The scores of clinical scales in the patients with CFS were higher than those in the controls (Table 1). NRG scores were decreased after the treatment compared to the pre-treatment values of the patients (Table 2).

The pre-treatment basal cortisol levels of the patients were significantly lower than those of the controls (F=4.129, p=0.049). No significant difference was found in serum DHEAS levels between the patients and the controls before the treatment (F=2.811, p=0.101), although the patients had elevated DHEAS/cortisol molar ratios compared to the controls (F=7.318, p=0.010). The cortisol values of the patients continued to be low after the treatment compared to the controls (F=4.803, p=0.035); however, the post-treatment basal DHEAS values and DHEAS/cortisol molar ratios were similar to those of the controls (F=0.153, p=0.698; F=3.026, p=0.090; respectively)(Table 3).

In the patient group, there was no significant difference in serum cortisol levels and DHEAS/cortisol molar ratio of the patients between before (mean±SD: 11.19±4.83 for cortisol, 136.32±90.34 DHEAS/cortisol molar ratio) and after (mean±SD: 10.71±3.93 for cortisol, 114.18±75.99 for DHEAS/cortisol molar ratio) the treatment, according to a repeated measure ANOVA test (F=1.012, p=0.328; F=0.546, p=0.470; respectively). After the treatment DHEAS levels (mean±SD: 1114.77±638.60) were significantly decreased compared to those before the treatment (mean±SD: 1536.37±1094.72)(F=6.723, p=0.018).

**FIGURE 1:**
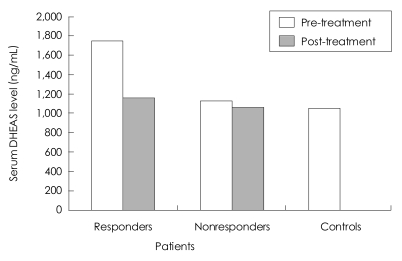
Basal serum DHEAS values of responders and non-responders before and after treatment and the controls. DHEAS: dehydroepiandrosterone sulfate.

When the CFS patients were separated as responders and nonresponders to the treatment, serum cortisol values of the two subgroups did not differ from those of the controls before or after the treatment. The pre-treatment DHEAS values and DHEAS/cortisol molar ratios of the responders were significantly higher than those of the controls (F=5.382, p=0.029; F=5.722, p=0.025 respectively). No significant difference was found in the pre-treatment DHEAS/cortisol molar ratios between the non-responders and the controls (F=3.627, p=0.067). There were no statistically significant differences in the pre-treatment DHEAS values and DHEAS/cortisol molar ratios between the responders and the non-responders (F=2.902, p=0.107; F=1.082, p=0.313 respectively). A comparison of the pre-treatment and the post-treatment hormone values demonstrated that the pre-treatment DHEAS values were only significantly higher than the post-treatment DHEAS values in responders (F=12.119, p=0.013)(Table 4)(Figure 1).

No statistically significant difference was found in the hormonal changes between the male patients and the female patients (Z=0.274, p=0.820 for delta cortisol, Z=1.528, p=0.140 for delta DHEAS, Z=1.293, p=0.218 for delta DHEAS/cortisol ratio).

A negative correlation was observed between the DHEAS values and age in the patient and control groups as expected (r=-0.601, p=0.001; r=-0.513, p=0.021; respectively). There were strong positive correlations between the changes in the DHEAS values (delta DHEAS) and those in scores of depression (delta HAM-D) and anxiety scores (delta HAM-A) in the responder group (r=0.831, p=0.003; r=0.790, p=0.007; respectively), but not in the non-responder group (r=0.393, p=0.207; r=0.525, p=0.080; respectively). The correlation between delta DHEAS and delta NRG scores was not statistically significant in the responder or non-responder groups (r=0.589, p=0.073; r=0.350, p=0.265; respectively). No correlation was found between the changes in DHEAS/cortisol and those in clinical scales.

## Discussion

The important findings of this study are: 1) the absence of any effect of the galantamine treatment on lowered cortisol levels in CFS patients, 2) higher basal DHEAS values and higher DHEAS/cortisol molar ratios which were normalized following four weeks' treatment with 8 mg/d galantamine hydrobromide in the treatment-respondent group.

### Hypocortisolism

Many studies of patients with CFS have found HPA axis dysregulation and hypocortisolism. In these studies cortisol levels were decreased in 24-hour urine,^7^ serial blood samples,^10^ or saliva,^11^ as seen in our study, but it is not clear whether low cortisol levels and HPA hypofunction in CFS are a cause or result of the illness. Taking into account the finding of this study of the reduced cortisol levels of the pre-and post-treatments, we can infer that HPA alterations in CFS are the persisting features of the disease, although we do not make any suggestions as to whether it is a cause or a consequence of the illness. Reduced HPA axis functioning^6^ has also been suggested to cause some of the symptoms, like reduced energy, post-exertional malaise, and headaches^1^ seen in CFS patients. In a previous study 5-10 mg/d hydrocortisone therapy resulted in a fall in fatigue scores of 34%.^33^ One explanation for hypocortisolism in CFS may be increased glucocorticosteroid receptor sensitivity.^34^

The finding of unchanged cortisol level after galantamine treatment of this study is consistent with a recent study by Blacker et al.^26^

How can we explain the finding of the study that while cortisol levels remained lower throughout the galantamine treatment some patients showed symptomatic improvement at partial level? It has been suggested that a functional deficiency of cholinergic neurotransmission may result in hypoactive HPA axis under stress conditions^35^ and may have a role at pathophysiology of CFS. Cholinergic neurotransmission stimulates growth hormone and insulin-like growth factor I secretions, which plays an important role in the regenerative functions of the peripheral nerves and skeletal muscles.^35^ Taking together these findings, increased cholinergic activity by galantamine treatment might trigger well-being independently of cortisol levels in some patients. This means, at least in some patients, that it may not be necessary to normalize cortisol levels for an improvement to be achieved. However, if we followed the patients in the long-term we could have a better idea about cortisol levels. As a conclusion we can suggest that in some patients with CFS there may be dysregulation in other systems like the cholinergic system along with the HPA axis.

### Dehydroepiandrosterone sulfate levels and dehydroepiandrosterone sulfate/cortisol molar ratios

Given the findings of lower pre- and post-treatment cortisol levels, higher pre-treatment DHEAS levels compared to post-treatment, and higher DHEAS/cortisol molar ratios in the patient group, we can suggest that there may be a disturbance in the 'metabolic shift' from androgens to glucocorticoids.

DHEA/cortisol or DHEAS/cortisol molar ratios were used in many studies as variants that reflected the homeostasis between androgens and glucocorticoids at adrenal level.^17^,^36^ Scott et al.^37^ performed a study in CFS patients without any psychiatric comorbidity. They found that DHEA and DHEAS levels were lower than in healthy controls and explained this finding with mild adrenal cortical atrophy (failure) secondary to ACTH deficiency. Scott et al.^37^ also did not identify any difference between CFS patients, depressives, and healthy controls with respect to basal cortisol levels. De Becker et al.^38^ demonstrated that basal DHEA levels were normal, but DHEA responses to ACTH stimulation were blunted in CFS patients. No cortisol levels were reported in that study. Cleare et al.^36^ found higher basal DHEA levels in CFS patients with no psychiatric comorbidity compared to healthy controls; however, they did not report any difference in DHEAS levels. In the same study, they did not find any difference between patients and controls with respect to cortisol/DHEA molar ratio. In addition, the investigators administered low dose (5-10 mg/d) hydrocortisone to CFS patients and determined significant decreases in DHEA levels in patients who reported declined fatigue scores with treatment. They found significant reductions in DHEAS after hydrocortisone treatment. Similarly, we also found decreased DHEAS levels after galantamine treatment in CFS patients. This may be interpreted as the fact that the acetylcholinergic effect of galantamine facilitates the release of CRH hormone and in turn, causes elevated DHEAS levels to decrease through recovering the existing tentative disturbance in metabolic shift from cortisol to DHEAS.

Some studies have reported low^39^,^40^ or normal^36^,^41^ morning DHEAS levels in CFS patients. An investigator reported a slightly high DHEA levels in CFS patients.^18^ Similarly, Goldberg and Lichten^42^ reported DHEAS levels above the reference limit in 20% of their patient group, made up of 140 females with CFS. In our study increased pre-treatment DHEAS/cortisol molar ratio may have been due to impaired HPA axis activity and decreased cortisol levels or a defect in metabolic shift from androgen to glucocorticoid production. It was proposed that the literature findings about higher cortisol/DHEA molar ratios in major depression were the most important indicators of excessive cortisol exposure of the brain.^43^,^44^ On the other hand, our finding of high DHEAS/cortisol molar ratios might lead to further decreases in the low cortisol effects on the brain. However, only basal levels were investigated in this study. Circadian measurements might provide further information on this subject.

The controversies in all these studies might be explained by the differences in clinical characteristics (psychiatric comorbidity, medicines), methodological discrepancies (differences in the timing of blood sampling or laboratory techniques), and other factors that might affect functions of the HPA axis, like inactivity. Our study had the advantages of including pure and untreated CFS patients.

### Responders and non-responders to treatment

When we separated our patients into two subgroups as responders and non-responders to treatment, the pre-treatment basal DHEAS levels and DHEAS/cortisol molar ratios of the responders were significantly higher than those of the controls; however, it was interesting to find the values of the non-responders similar to those of the controls. To our knowledge, these findings are unique in the literature. It was concluded that high levels of pre-treatment DHEAS activity and its ratio to cortisol might be an indicator of the responsiveness to treatment.

It is possible to explain the high pre-treatment basal DHEAS levels and DHEAS/cortisol molar ratio in responders and the decrease in DHEAS levels with galantamine hydrobromide treatment in all CFS patients and especially in responders in several ways. The first is that high pre-treatment DHEAS levels and DHEAS/cortisol molar ratio in responders may be due to hypocortisolism supposed to be present in CFS. Improvement in relative hypocortisolism with treatment may provide a return to the normal DHEAS pattern. However, we did not observe any significant increase in cortisol levels. A longer period might be needed for such a finding. The second possible explanation is that some abnormalities in the metabolic pathway of DHEAS like 21-hydroxylase enzyme deficit^42^ may lead to higher pre-treatment DHEAS synthesis. The third is that normalization of high DHEAS levels in responders may be secondary to improvement in some clinical features such as sleep disturbances, anxiety or some depression symptoms which may be available in these patients.^45^ Indeed, the finding of the present study of a positive correlation between reduction of the DHEAS levels with galantamine treatment and recovery in clinical depression and anxiety scales in only respondent patients supports this hypothesis strongly. The fourth is that abnormal glucocorticoid receptor sensitivity may improve with treatment and the production may shift from DHEAS to cortisol. Finally, the cholinergic effect of galantamine that facilitates release of CRH may cause reduction in DHEAS levels and the normalization of high DHEAS/cortisol molar ratio. We think that the duration of our experimental treatment may not have been sufficient to show these changes.

There are several limitations of the study. A small number of patients and the absence of placebo control group might have prevented us from observing the therapeutic and hormonal effects of the galantamine more accurately. Additionally, the short duration of the treatment might obscure the long-term effects of the drug on fatigue scores or on the hormones such as cortisol.

In conclusion, decreases in basal DHEAS levels and DHEAS/cortisol molar ratios in CFS patients with galantamine treatment may somewhat support the hypothesis that cholinergic deficit may be present in CFS. On the other hand, DHEAS findings of responders suggest that high pre-treatment values of this hormone may be an indicator of the treatment responsiveness. If DHEA and DHEAS levels play an important role in CFS physiopathology, we think that, in addition to the employment of non-invasive techniques like the measurement of cortisol levels in the saliva, the evaluation of DHEA (-S) levels with response to naturalistic stressors (challengers) like exercise or social stress in future studies will be beneficial.

## Figures and Tables

**TABLE 1:**
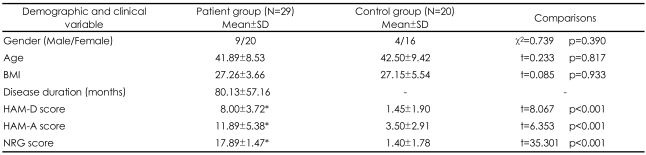
Socio-demographic and clinic characteristics of the patient and control groups

^*^Significantly higher than those of the controls. n: number of subjects, BMI: Body Mass Index, HAM-D: Hamilton Depression Rating Scale, HAM-A: Hamilton Anxiety Rating Scale, NRG: Newcastle Research Group ME/CFS Score Card

**TABLE 2:**
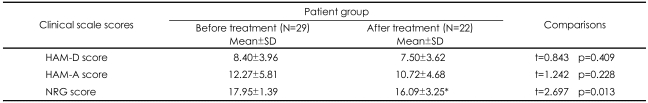
Comparison of clinical scale scores of the patients before and after the treatment

^*^Significantly lower than those in pre-treatment values. HAM-D: Hamilton Depression Rating Scale, HAM-A: Hamilton Anxiety Rating Scale, NRG: Newcastle Research Group ME/CFS Score Card

**TABLE 3:**
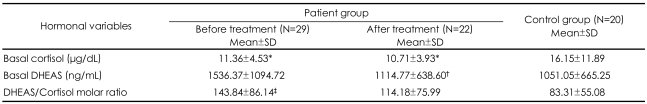
Hormonal values before and after treatment in the patient and control groups

^*^Significantly lower than that of the control group (F=4.129, p=0.049; F=4.80 3, p=0.035), ^†^Significantly lower than before the treatment (F=6.723, p=0.018), ^‡^Significantly higher than that of the control group (F=7.318, p=0.010). DHEAS: Dehydroepiandrosterone sulfate

**TABLE 4:**
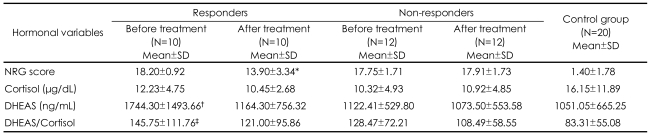
Hormonal values of responders and non-responders before and after treatment

^*^Lower than pre-treatment (t=4.502, p=0.001), ^†^Higher than post-treatment and controls (F=12.119, p=0.013; F=5.382, p=0.029), ^‡^Higher than controls (F=5.722, p=0.025). NRG: Newcastle Research Group ME/CFS Score Card, DHEAS: dehydroepiandrosterone sulfate
